# Oesophageal submucosal hematoma after flow diverter embolization with favourable outcome treated by discontinuing postoperative antiplatelet therapy for only three days: a case report

**DOI:** 10.1186/s40981-018-0222-x

**Published:** 2019-01-08

**Authors:** Eriko Takeyama, Aiko Wada, Eizo Amano, Hiromi Shibuya

**Affiliations:** 1 Department of Anesthesiology, National Hospital Organization Osaka National Hospital, 2-1-14, Hoenzaka, Chuo-Ku, Osaka-city, Osaka, 540-0006 Japan; 2 Department of Anesthesiology and Intensive Care Medicine, Graduate School of Medicine, Osaka University, 2-2, Yamada-oka, Suita, Osaka, 565-0871 Japan

**Keywords:** Oesophageal submucosal hematoma, Endovascular surgery, Antiplatelet therapy

## Abstract

**Background:**

Oesophageal submucosal hematoma is a rare perioperative complication. When this complication develops after endovascular surgery, which requires postoperative antiplatelet therapy, whether to stop antiplatelet therapy or not is controversial. If antiplatelet therapy is discontinued, the appropriate time to resume antiplatelet therapy is unclear.

**Case presentation:**

A 75-year-old woman (height 134 cm, weight 37 kg) underwent flow diverter embolization for unruptured cerebral aneurysm under general anaesthesia. The patient received dual antiplatelet therapy before surgery and anticoagulation therapy intraoperatively. After surgery, the patient developed hematemesis and was diagnosed with oesophageal submucosal hematoma. Conservative treatment was initiated after discontinuing antiplatelet therapy, which was resumed 3 days after surgery. The patient showed good recovery even after the resumption of antiplatelet therapy.

**Conclusions:**

In our case, we successfully treated oesophageal submucosal hematoma developing after endovascular surgery with early resumption of postoperative antiplatelet therapy.

## Background

Oesophageal submucosal hematoma is a rare perioperative complication. There have been several reports of this complication after endovascular surgery, which requires perioperative antiplatelet therapy. For these cases, physicians have to treat patients considering both the management of haemorrhage associated with the oesophageal submucosal hematoma and the prevention of thromboembolic complications after endovascular treatment. We report a case of oesophageal submucosal hematoma after placement of a flow diverter, a new-generation endovascular device for unruptured cerebral aneurysm-associated long-term postoperative antiplatelet therapy to prevent thromboembolic complications. We treated the patient successfully by suspending the antiplatelet therapy temporally and resuming it 3 days after hematoma development. To the best of our knowledge, this is the first report of a patient with oesophageal submucosal hematoma resuming antiplatelet therapy after only 3 days of suspension.

## Case presentation

A 75-year-old woman (height 134 cm, weight 37 kg) underwent flow diverter placement for an unruptured cerebral aneurysm under general anaesthesia. Her medical history and preoperative complications were unremarkable. Preoperative laboratory data were within normal limits, with no coagulation abnormalities. She received dual antiplatelet therapy with aspirin and clopidogrel beginning 10 days prior to surgery.

Anaesthesia was induced with a target-controlled infusion of propofol (3.0 μg/ml) and fentanyl (50 μg). After administration of rocuronium (30 mg), tracheal intubation was performed uneventfully with a video laryngoscope (McGRATH MAC™), followed by smooth insertion of a gastric tube. The gastric tube was maintained without suction intraoperatively. Anaesthesia was maintained with target-controlled infusion of propofol and remifentanil 0.05–0.1 μg/kg/min with an inhaled oxygen concentration of 42% under standard monitoring, as well as direct radial artery pressure monitoring.

Heparin (4000 units) was intravenously administered at the beginning of surgery. Activating clotting time was 159, 254, and 244 s before surgery, 15 min after starting surgery, and at the end of surgery, respectively. The operative approach was from the femoral artery. Surgery was performed without complications. Protamine was not administered at the end of surgery. Intraoperatively, the patients’ blood pressure remained at 80–120/40–50 mmHg and her heart rate remained at 55–65 beats/min with no acute hemodynamic changes.

Continuous infusion of propofol and remifentanil were discontinued at the completion of surgery. The patient awakened 20 min after completion of surgery, and her systolic blood pressure increased to 150 mmHg. The gastric tube was removed without any abnormal findings, such as blood in the secretions. The tracheal tube was removed smoothly. Continuous infusion of argatroban was initiated at completion of surgery. The operation time was 97 min, and anaesthesia time was 172 min.

**Fig. 1:**
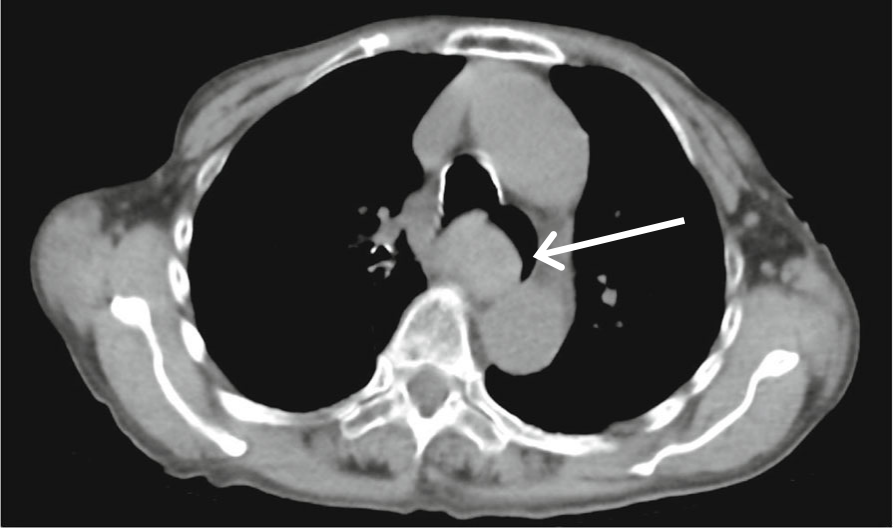
Computed tomography thoracic examination immediately after hematemesis. The thoracic oesophagus is dilated. The white arrow indicates the oesophagus

**Fig. 2:**
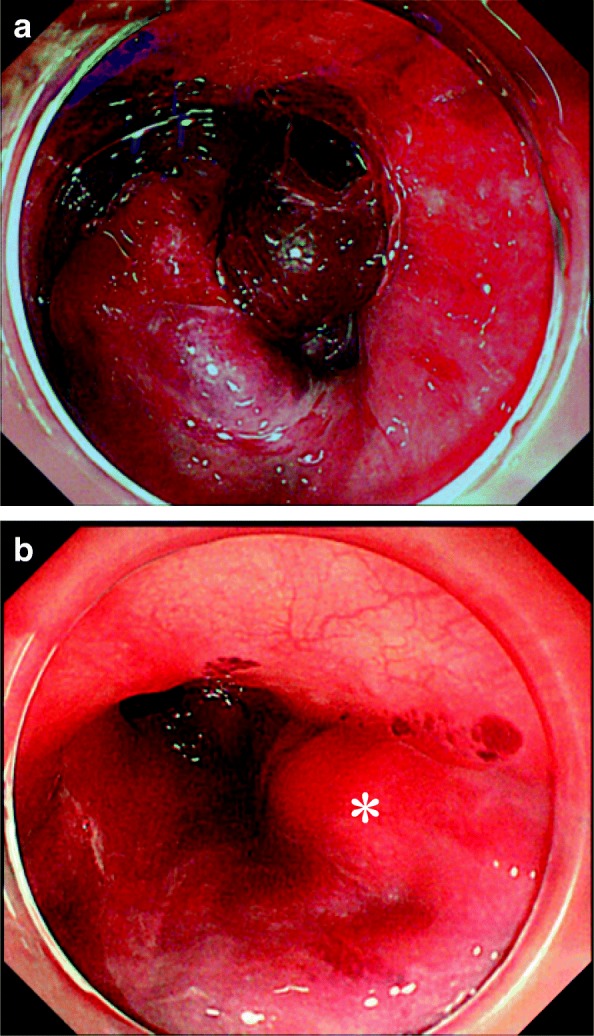
Upper gastrointestinal endoscopy images after hematemesis. **a** The submucosal hematoma occupied the oesophagus. **b**, **a** Longitudinal extension of reddish or wine-coloured mucosal thickening (asterisk), obstructing the oesophagus, is seen from the cervical oesophagus to the thoracic oesophagus

Due to systolic blood pressure greater than 150 mmHg, continuous infusion of nicardipine was initiated to maintain systolic pressure below 140 mmHg after entering the recovery room. The patient started to complain of chest pain 90 min after entering the recovery room. At that time, her blood pressure was 155/77 mmHg and her pulse rate was approximately 70 bpm. The electrocardiogram was unremarkable, and blood biochemical haemostatic function testing showed no abnormal findings without prolonged activated partial thromboplastin time (120 s). She developed hematemesis 120 min after entering the recovery room. Her blood pressure and heart rate remained stable. Emergent computed tomography (CT) was performed. Although the vascular system was normal, expansion of the oesophagus was noted (Fig. 1). Due to a decrease in blood pressure to 90/47 mmHg and haemoglobin level from 11.7 to 8.7 g/dL, the patient was given fluid resuscitation and was transfused with 6 units of red blood cells and 6 units of fresh frozen plasma following CT examination. Considering the risk of tracheal aspiration, we performed tracheal intubation. Upper gastrointestinal endoscopy (UGE) was performed, and an oesophageal submucosal hematoma extending longitudinally on the right wall, 17 cm from the incisor, was noted. Because a narrowed lumen and oozing due to scope contact were recognised, observation was limited to the lower oesophagus (Fig. 2).

Conservative treatment was initiated with the cessation of continuous infusion of argatroban and the planned postoperative antiplatelet therapy. CT performed 2 days postoperatively showed reduced dilation of the oesophagus, and thus, the tracheal tube was removed. A nasogastric tube was inserted 3 days after surgery without any abnormal findings, and antiplatelet therapy was resumed on the same day. The patient had no symptoms even after the resumption of antiplatelet therapy. UGE performed 11 days postoperatively showed that the submucosal hematoma was almost completely resolved, and solid food intake was resumed. The patient showed good recovery and was discharged 27 days after surgery.

## Discussion

Oesophageal submucosal hematoma is a rare condition. The spontaneous type of oesophageal submucosal hematoma is considered to be caused by the rupture of blood vessels in the submucosal layer [1], as the result of a sudden increase in pressure induced by factors such as nausea and vomiting. It is also suggested that oesophageal submucosal hematoma is associated with bleeding tendency, such as antiplatelet or anticoagulant therapy [2]. Although reports of intraoperative oesophageal submucosal hematoma are rare, a few cases have been reported of this complication after endovascular surgery for unruptured cerebral aneurysms. Endovascular surgery requires perioperative antiplatelet therapy, which could be associated with the development of oesophageal submucosal hematoma. The prognosis of this complication is generally good, and approximately 90% of reported cases receiving antiplatelet therapy had been treated conservatively with cessation of postoperative antiplatelet therapy [3–9].

The flow diverter is a new-generation stent placed in the parent artery at the level of the aneurysm neck to disrupt inflow into the aneurysm sac and, thus, favour intra-aneurysm thrombosis. It is clinically used for large cerebral aneurysms that are difficult to treat with coil embolization. Conversely, long-term postoperative antiplatelet therapy is necessary to prevent severe thromboembolic complications, since a prolonged time period is required for aneurysm resolution. However, there are no clear criteria for drug selection, administration period, and short-term discontinuation of antiplatelet therapy due to haemorrhagic events.

Therefore, oesophageal submucosal hematoma after flow diverter placement must be treated considering not only the risk of bleeding but also the possibility of thromboembolic complications.

Considering the risk of bleeding, the risk of potentially severe oesophageal submucosal hematoma should be considered. Although this complication often shows a good prognosis, cases of haemorrhagic shock associated with the onset of oesophageal submucosal hematoma have been reported [4]. In our case, the patient developed massive bleeding requiring transfusion. As the guideline for flow diverter use recommends cessation of antiplatelet therapy when a severe bleeding event occurs, discontinuation of antiplatelet therapy immediately after the development of this complication can be considered reasonable.

After cessation of antiplatelet therapy, the optimum timing to resume treatment is a serious concern due to the possibility of thromboembolic complications. While there are no broadly accepted practice patterns addressing dual antiplatelet therapy in the flow diversion literature, the length and dose of antiplatelet therapy are reported to correlate with a decreased incidence of thrombotic and haemorrhagic events after flow diverter placement [10]. In our case, we diagnosed haemostasis at a relatively early stage by CT examination and resumed treatment 3 days after surgery. Even after the resumption of antiplatelet therapy, the patient did not show relapse of symptoms and had a favourable course. In almost every reported case treated conservatively, hematoma contraction has been confirmed by CT or upper gastrointestinal endoscopy within 4 to 7 days after onset. These reports may suggest that active bleeding stops at this time point and that resumption of antiplatelet therapy may be considered.

In conclusion, the risk of oesophageal submucosal hematoma development after endovascular surgery should be considered. When postoperative antiplatelet therapy is necessary, such as flow diversion, it may be beneficial to perform periodic reassessments for haemostasis or oesophageal submucosal hematoma, and early resumption of antiplatelet therapy may be considered with careful observation.

## Abbreviations

CT: Computed tomography
UGE: Upper gastrointestinal endoscopy
